# PI3Kδ过度活化综合征一例

**DOI:** 10.3760/cma.j.issn.0253-2727.2021.11.016

**Published:** 2021-11

**Authors:** 博科 杨, 涛 吴, 亚琴 安, 东锋 毛, 玲玲 鱼, 宗慧 王, 海 白

**Affiliations:** 解放军联勤保障部队第九四〇医院（原兰州军区兰州总医院）全军血液病中心，兰州 730050 The 940th Hospital of Joint Logistics Support Force of Chinese People's Liberation Army, Lanzhou 730050, China

患儿，女，8岁，因“反复腹泻，间断乏力2年余，脾大”于2019年2月就诊。患儿2015年起出现反复腹泻症状，2017年患儿因乏力伴腹泻就诊于外院，B超示脾大。入院查体：贫血貌，腹部膨隆，脾脏肋缘下平脐，无压痛，肋缘下锁骨中线平脐、质韧、边钝，余腹部查体未见明显异常。血常规：WBC 5.75×10^9^/L、RBC 2.91×10^12^/L、HGB 86 g/L、PLT 122×10^9^/L。免疫球蛋白IgG<330 mg/L、IgA 655 mg/L、IgM 22.7 g/L。大便隐血阳性。腹部B超：肝大、脾大、副脾、腹腔淋巴结肿大。免疫固定电泳：λ轻链阳性。骨髓象：有核细胞增生明显活跃，细胞内铁53％。血游离轻链：κ轻链51 mg/L（参考值6.7～22.4 mg/L）、λ轻链 37.6 mg/L（参考值8.3～27.0 mg/L）；尿游离轻链：κ轻链 404 mg/L（参考值0～25.8 mg/L）、λ轻链278 mg/L（参考值0～11.3 mg/L）。红细胞渗透脆性试验：孵育前4.75 g/L，孵育后6.25 g/L。胸部CT：右肺中叶及两肺下叶亚段性不张支气管肺炎，纵隔、两侧腋窝及锁骨上窝多发淋巴结肿大，前纵隔不规则软组织影，考虑未退化的胸腺，两侧胸膜局限性增厚粘连，肝脏增大。行脾脏切除手术，术后脾脏病理结果示：（脾、肠系膜淋巴结）形态学改变结合免疫组化染色结果及临床（巨球蛋白血症），初步考虑淋巴浆细胞性淋巴瘤，MYD88基因突变检测阴性。脾脏病理送北京友谊医院会诊：脾脏髓外造血、淋巴结淋巴组织不典型增生。术后3个月患者便水样便，乏力，伴头痛、发热，无恶心呕吐、无腹痛、无咳嗽、咳痰。血常规：WBC 48.68×10^9^/L、HGB 115 g/L、PLT 798×10^9^/L。免疫球蛋白IgG 330 mg/L、IgA 723 mg/L、IgM 13.1 g/L。血培养检出肺炎链球菌。多次便培养检出肺炎克雷伯菌、极少量沙门菌，予头孢曲松抗感染、补液等治疗好转。复查血常规WBC进行性下降，考虑高IgM综合征可能性大。输注丙种球蛋白5 g治疗2次。遗传性疾病全外显子测序示：PIK3CD基因c.2974G>A（E992K）杂合突变，SPTB基因1个杂合意义未明突变。获一代测序验证。患者父亲无以上两个突变，母亲携带PIK3CD基因的杂合突变，不携带SPTB基因的杂合突变。考虑患者PIK3CD基因的杂合突变遗传自母亲，SPTB基因的杂合突变为新发突变。诊断：PI3Kδ过度活化综合征（APDS）。予氟达拉滨+白消安+环磷酰胺+抗胸腺细胞球蛋白方案预处理后行单倍型造血干细胞移植，环孢素A+霉酚酸酯+短程甲氨蝶呤联合预防移植物抗宿主病。+13 d粒系、血小板植入。全血嵌合69.13％，B细胞67.73％，T细胞72.48％，NK细胞85.32％，表现为混合嵌合状态。+60 d复查血常规：WBC 15.00×10^9^/L、HGB 92 g/L、PLT 183×10^9^/L，免疫球蛋白IgG 3370 mg/L、IgA <67 mg/L、IgM 285 mg/L，骨髓呈完全嵌合状态。+106 d复查血常规：WBC 10.51×10^9^/L、HGB 102 g/L、PLT 204×10^9^/L，全血嵌合99.77％，NK细胞98.85％，B细胞99.71％，表现为完全嵌合状态。目前仍在后续治疗随访中。

讨论：APDS是一种由PIK3CD基因突变导致的原发性免疫缺陷病（primary immunodeficiency disease，PID），主要临床表现为反复呼吸道感染、良性淋巴组织增生、巨细胞病毒和（或）EB病毒血症等。PIK3CD基因的热点突变为c.3061G>A（E1021K）点突变，国内已报道的患者均为该位点新生突变，本例患儿为PIK3CD基因的1个杂合致病突变c.2974G>A（E992K），遗传自其母亲。APDS的临床表现差异较大，部分患者无明显症状，部分在儿童期就出现严重的免疫缺陷。APDS免疫表型具有可变性，所以目前尚无统一的临床诊断标准，主要依靠基因检测确诊。临床该病容易误诊为高IgM综合征、X连锁淋巴组织增生综合征以及其他原发性免疫缺陷病。

